# Efficacy of Selective Arterial Embolisation for the Treatment of Life-Threatening Post-Partum Haemorrhage in a Large Population

**DOI:** 10.1371/journal.pone.0003819

**Published:** 2008-11-26

**Authors:** Cyril Touboul, Wassim Badiou, Julien Saada, Jean-Pierre Pelage, Didier Payen, Eric Vicaut, Denis Jacob, Arash Rafii

**Affiliations:** 1 Department of Obstetrics and Gynaecology, Hôpital Lariboisière, AP-HP, Paris, France; 2 Department of Vascular Imaging, Hôpital Lariboisière, AP-HP, Paris, France; 3 Department of Anaesthesiology and Critical Care, Hôpital Lariboisière, AP-HP, Paris, France; 4 Department of Nuclear Medicine, Hôpital Lariboisière, AP-HP, Paris, France; 5 Department of Genetic Medicine and Obstetrics and Gynecology, Weill Cornell Medical College, Paris, France; Khon Kaen University, Thailand

## Abstract

**Background:**

The objective of this study was to assess efficacy and determine the optimal indication of selective arterial embolisation (SAE) in patients with life-threatening post-partum haemorrhage (PPH).

**Methodology/Principal Findings:**

One hundred and two patients with PPH underwent SAE and were included from January 1998 to January 2002 in our university care center. Embolisation was considered effective when no other surgical procedure was required. Univariate and multivariate statistical analysis were performed. SAE was effective for 73 patients (71.5%), while 29 required surgical procedures. SAE was effective in 88.6% of women with uterine atony that was associated with positive outcome (OR 4.13, 1.35–12.60), whereas caesarean deliveries (OR 0.16, 0.04–0.5) and haemodynamic shock (OR 0.21, 0.07–0.60) were associated with high failure rates, 47.6% and 39.1%, respectively.

**Conclusions/Significance:**

Success rate for SAE observed in a large population is lower than previously reported. It is most likely to succeed for uterine atony but not recommended in case of haemodynamic shock or after caesarean section.

## Introduction

Post-partum haemorrhage (PPH) occurs in 2–11% of deliveries, remains a major cause of maternal mortality and morbidity, despite advances in obstetrics and intensive care.[1], [2] In developed countries, life-threatening PPH occurs in 1 per 1000 deliveries;[3]–[5] it is the main cause of maternal mortality in France, responsible for more than 20 of the 80 maternal deaths each year [1], [2]. Prevention and rapid identification of the source and control of the bleeding are essential to prevent maternal morbidity and mortality [6], [7]. In most cases PPH can be managed medically with such uterotonic drugs as oxytocin or prostaglandin analogues [4], [8]. When medical treatment remains insufficient, three therapeutic options are available. The two standard alternatives are vascular ligature (e.g., of the hypogastric or uterine arteries) [3], [9], with success rates ranging from 40% to 100% [10]–[15], and emergency hysterectomy, which is the treatment of last resort for PPH [3]. Selective arterial embolisation (SAE) of the uterine arteries was recently proposed as an alternative to surgery, and success rates for controlling severe PPH with it are reported to range from 85% to 95% [13], [16]. Its benefits seem to include a low complication rate and the avoidance of the risks of surgery and general anaesthesia in haemodynamically unstable patients [13], [17].

The studies evaluating SAE so far, however, have two major methodological drawbacks: relatively few patients (9 to 35) and imprecise descriptions of the severity of the PPH and of the patients' general status [13], [16].

The aim of our study was to assess the efficacy and determine the optimal indication for SAE in the management of severe life-threatening PPH in a large population.

## Methods

### Objectives

The objective of our study was to assess the efficacy and determine the optimal indication for SAE in a large population of patients with severe life-threatening PPH.

### Participants

This study included all women who had life-threatening primary PPH and underwent SAE in our university teaching hospital from January 1998 through January 2002. They either gave birth in our obstetrics department or were transferred from other institutions that did not have either an intensive care unit or a vascular imaging unit. Severe PPH was defined as a blood loss greater than 1500 cc and either haemodynamic shock (defined by the need for continuous perfusion of vasopressors) or disseminated intravascular coagulation (platelet count <50 000 per mm^3^, elevated prothrombin time, defined as greater than twice the control values, hypofibrinogenemia, defined as less than 150 mg/dl, and a prothrombin rate less than 50%), or both. Blood loss was estimated by collection bag which was systematically placed at the end of the delivery. For the patients transferred from other institutions, we added the estimated blood loss evaluated by the medical team of the hospital of origin with the estimated blood loss in our hospital.

### Description of Procedures or Investigations undertaken

**Figure 1:**
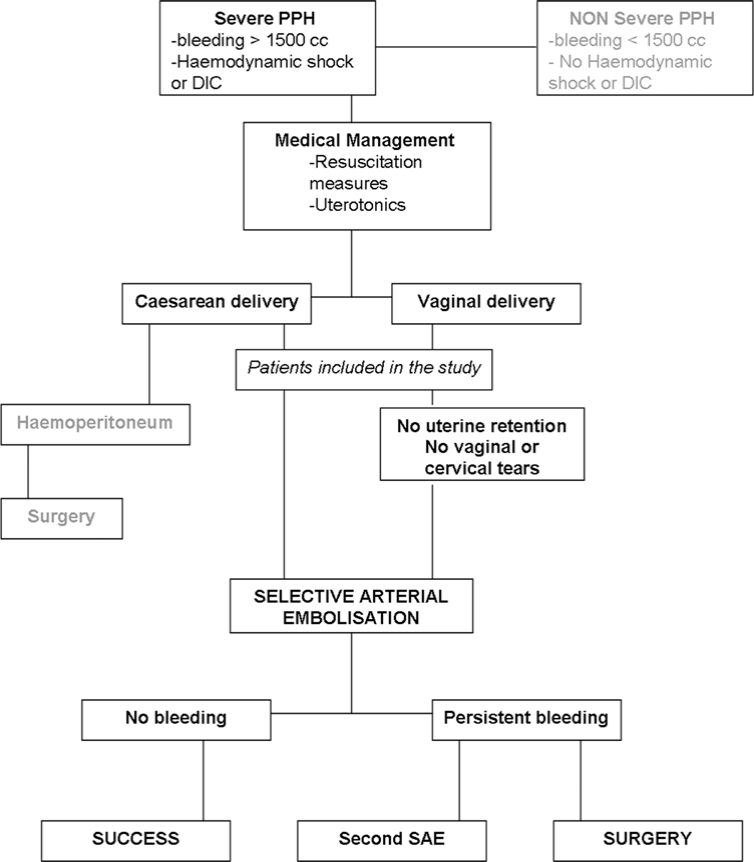
Decision algorithm for treatment of post-partum haemorrhage by selective arterial embolisation in our institution.

All patients were treated according to the same treatment protocol for PPH, summarised in the decision algorithm approved by the three departments involved in this study (Figure 1).

Anaesthesiology management in the intensive care unit included initial evaluation and resuscitation. Patients were monitored with continuous oximetry, heart rate and blood pressure, indwelling urinary catheter for hourly urine output measurements, and femoral venous and arterial catheters. Resuscitation involved fluid (colloids and crystalloids) replacement and blood and plasma transfusion responsive to laboratory results. In case of uncontrolled hypotension, continuous norepinephrine infusion was started on the femoral line. Mechanical ventilation was initiated in case of uncontrolled haemorrhagic shock with neurological failure. For sedation we used midazolam and sufentanil or ketamine in case of severe shock. Amoxicillin and clavulanic acid were systematically administered for antibiotic prophylaxis.

Basic obstetrical management for patients with vaginal delivery involved bimanual uterine examination, removal of any retained placental parts or membranes, and careful inspection of the genital tract for lacerations or tears. Any surgical tears was repaired before performing the SAE. Patients who had caesarean deliveries underwent abdominal ultrasound to verify the absence of retained placental pieces in the uterus and to rule out hemoperitoneum. Medical management included uterine massage, intravenous diluted oxytocin up to a cumulative dose of 55 IU (Syntocinon; Sandoz, Reuil-Malmaison, France) and sulprostone, a prostaglandin-E2 analogue (a first injection of 500 µg over an hour and a second injection of 500 µg over four hours) (Nalador; Schering, Lys-les-Lannoy, France).

After obstetrical evaluation patients were transferred under close monitoring to the vascular imaging department for SAE. Resuscitation continued during SAE. The vascular radiologist performed emergency digital subtraction angiography with an unilateral femoral approach. In most cases contralateral internal iliac angiography and selective study of the anterior division then followed, always with a 5-F cobra catheter and a hydrophilic polymer-coated 0.032-inch guide wire (Radiofocus; Terumo) to analyse the uterine arteries. Super-selective study of the uterine artery was attempted in all cases. Other anastomotic vessels, including the vaginal, ovarian and round ligament arteries, were studied when necessary. The ipsilateral internal iliac artery was then catheterised by the same puncture site and the same cobra catheter. Absorbable gelatin sponge pledgets were injected as embolisation agents under fluoroscopic guidance. Bilateral embolisation was attempted in all patients. When the uterine artery could not be selectively catheterised, the anterior division of the hypogastric artery was embolised instead. Post-embolisation angiography was performed to verify the complete occlusion of the vessels.

All patients were transferred to the surgical intensive care unit for at least 24 hours for further observation and coagulation studies with the femoral artery sheath left in place until achievement of haemodynamic stability, correction of coagulation disorders and complete cessation of the haemorrhage.

In this study we considered SAE effective when bleeding stopped after the procedure. The embolisation was considered ineffective when any additional surgical procedure was necessary to stop the bleeding completely.

Data extracted from the patients' records included individual and obstetric characteristics (maternal age, parity, mode and term of delivery, cause of PPH), as well as those relevant to critical care, haemostasis and coagulation (shock, disseminated intravascular coagulation, mean haemoglobin level, mean platelet count, mean prothrombin time, mean partial thromboplastin time expressed as ratio to control values, mean number units of whole blood, fresh frozen plasma and platelets transfused).

### Ethics

The French Research Ethics Committee chair stated that approval for this study was not required.

### Statistical methods

Results are expressed as means±SE. We used univariate procedures (Student's t test or chi-square test, as appropriate) to test possible associations of laboratory findings and clinical parameters with the embolisation results. We first analysed the relation between the patients' characteristics (e.g., causes of PPH) and SAE results and then that between the critical care-related variables and SAE outcome. To estimate the independent predictive value of each variable, they were all analysed in a multivariate procedure with stepwise logistic regression (Biomedical Data Processing Package, University of California, Los Angeles, California). Conservative criteria were used to select variables (p value of 5% required for entry into or removal from model) and the ratio between the regression coefficient for each factor and its standard error had to exceed 2.

## Results

During the study period 102 patients were treated with SAE and enrolled in the study: 12 (11.7%) gave birth in the obstetrics department of our institution and 90 (88.3%) patients were transferred from other obstetrics units.

### Patients' Characteristics

Table 1 reports the women's obstetrical characteristics. PPH was caused by uterine atony (N = 44, 43.1%), cervical or vaginal tears (N = 20, 19.6%), abnormal placentation (including placenta accreta and percreta) (N = 14, 13.6%), vaginal thrombus (N = 11, 10.7%), intrauterine retention (N = 7, 6.8%), abruptio placentae (N = 4, 3.9%) and repaired uterine rupture, (N = 2 , 1.9%).

**Table 1:** Maternal and obstetrical characteristics of the patients.

Age	
Mean±Standard Deviation	31.8 years±5.9
Range	21–45 years
Parity	
Mean±Standard Deviation	2.01±1.11
Range	1–6
Term of Delivery	
Mean±Standard Deviation	38.3±2,9 weeks
Range	28 to 42 weeks
Multiple pregnancies (twin) n (%)	4 (3.9%)
Mode of delivery	
Vaginal delivery n (%)	82 (79.4%)
- Forceps delivery n (%)	28/81 (34.5%)
Caesarean delivery n (%)	22 (20.6%)
Birth Weight	
Mean±Standard Deviation	3263.4±546.6
Range	2120–4751

Overall, 46 (45%) patients had haemodynamic shock, and 59 (57.8%) disseminated intravascular coagulation. At admission, the mean haemoglobin level was 6.03±1.6 dg/l (1.8–10.5), mean platelet count 81 698±46 740 (28 000–240 000), mean prothrombin rate 42.7±18.3 (5–90) and mean partial thromboplastin time (relative to control values) 2.15±1.8 . The mean number of units of whole blood transfused was 8.6±6.3 (1–31), of fresh frozen plasma 3.5±4.3 (0–23) and of platelets 1.02±3.5 (0–14) respectively. Three women required emergency management of cardiac arrest.

SAE of both uterine arteries was performed in 57 (55.8%) cases, SAE of one uterine artery and embolisation of the contralateral anterior branch of the hypogastric artery in 24 (23.5%), and bilateral embolisation of the anterior branch of the hypogastric artery in the other 21 (20.5%). Other vessels were embolised in six (5.8%) women: 4 (3.9%) the cervicovaginal arteries, and 1 each (0.8%) the ovarian anastomosis and the round ligament artery.

Two women died during in this study. The first patient was 29 years old, gravida 7, para 3, with past medical history of pre-eclampsia (in 5 previous pregnancies) and intrauterine death (in 3). Caesarean delivery took place under general anaesthesia at 28 weeks' gestation, for severe pre-eclampsia. She was transferred to our surgical intensive care unit, after C-section, for persistent diffuse bleeding. At admission, her haemoglobin level was 8.4 dg/l, her platelet count 63 000, prothrombin rate 24%, and fibrinogen 35 mg/dl. Major shock followed, with bilateral mydriasis; haemoglobin dropped to 4.9 dg/l. She received 6 units of whole blood, 4 of fresh frozen plasma, and 4 of platelets and was transferred to radiology department for SAE. Her haemodynamic status remained stable under continuous perfusion of norepinephrine during and after SAE. At this point computed tomography of the brain showed a cerebral haemorrhage with intraventricular bleeding, although gynaecological bleeding had stopped by then. After a day in the ICU, two electroencephalograms revealed no cerebral activity; she died the next day.

The second patient, aged 38 years, gravida 4, para 4, had vaginal delivery followed by PPH due to cervicovaginal tears, that persisted despite immediate surgical repair and vaginal packing. Cardiorespiratory arrest ensued three hours later, requiring resuscitation and transfusion of 6 whole blood units. After transfer to our department, electrocardiac activity continued but circulatory activity was insufficient. We performed SAE of left uterine artery and right anterior branch of the hypogastric artery. Because of continued bleeding and haemodynamic instability, a hysterectomy followed, with the vaginal tear repaired during aortic cross-clamping. While bleeding stopped after surgery, severe multiorgan failure had already occurred, and she died 30 hours after delivery.

### Outcome of SAE

SAE was effective for 73 patients (71.5%), 14 (13.7%) of whom required a second embolisation during the first 24 hours. The other 29 women required surgery. These included 9 laparotomies with focused limited haemostasis, 7 hysterectomies, 2 uterine artery ligations, and 11 genital tear repairs. In these latter procedures SAE was performed after reparation of any genital tears however 11 patients had still persistent bleeding after initial repair and SAE thus requiring a second surgery. SAE led to a reduction of pelvis blood flow that might have increased the efficiency of secondary surgery.

General complications included cardiogenic pulmonary oedemas related to the haemorrhage in 5 women, transient renal failure in 7, one of whom subsequently developed cortical necrosis and end-stage renal failure, and myocardial ischaemia in 3. Local complications included ischaemia of the lumbar plexus in one patient, and gluteal pain persisting 4 months in another. The mean duration of stay in the intensive care unit was 2.07±1.2 days (1–6.2).

The factors significantly associated to embolisation success or failure are represented in Table 2. The univariate analysis showed that mode of delivery was correlated with successful SAE: it was successful for 63 of 81 (77.7%) with vaginal deliveries, compared with 11 of 21 patients with caesarean births (52.7%) (p = 0.017). We also analysed the role of different causes of PPH on the outcome of SAE: uterine atony was associated with high rate of success (39 of 44 patients, 88%) (p<0.005). Efficacy of SAE did not differ according to the other causes, but vaginal and cervical tears had a trend towards lower success rates: 9 failures in 20 patients (45%)(p = 0.06). Of the other characteristics considered, failure was significantly associated with shock (18 failures among 46 patients, 39.1%, p = 0.02).

**Table 2:** Factors significantly associated to embolisation success or failure.

Factors	Successful embolisation (N = 73)	Unsuccessful embolisation (N = 29)	P (CI)
Vaginal delivery	63/73 (86.3%)	18/29 (62%)	0.01
Caesarean	11/73(15%)	10/29 (34%)	0.01
Uterine atony	39/73 (53%)	5/29 (17.2%)	<0.001
Vaginal and/or cervical tears	11/73 (15%)	9/29 (31.%)	0.06
Haemodynamic shock	28/73 (38.3%)	18/29 (62%)	0.02

When we used multivariate analysis to examine factors that predicted success of arterial embolisation, three variables were significant: good outcome was associated with uterine atony (odds ratio (OR 4.13 (95% CI, 1.35–12.6)), while poor outcome was associated with caesarean delivery (OR 0.16 (95% CI, 0.04–0.5)) and with shock (OR 0.21 (95% CI, 0.07–0.6)).

## Discussion

Efficiency of SAE was 71.5% in the whole study population. Uterine atony that was associated with positive outcome with an overall success rate of 88.6% (OR 4.13, 1.35–12.60). However, caesarean deliveries (OR 0.16, 0.04–0.5) and haemodynamic shock (OR 0.21, 0.07–0.60) were associated with higher failure rates, 47.6% and 39.1%, respectively.

SAE is a non-surgical, minimally invasive method of uterine devascularisation [13]. Over the past five years, this technique has become widely used in the treatment of PPH in France [16], [18]. Previous studies have reported low morbidity and excellent success rates for SAE, ranging from 85% to 95% [13], [16], [18]. The literature so far nonetheless has several limitations. Most of the studies are retrospective, and the two largest prospective studies include only 27 and 35 patients [16], [18]. The 71% success rate we report here is lower than the rates reported in previous studies. We included all patients with severe life-threatening PPH. All these patients were admitted to the intensive care unit because of haemodynamic instability or severe coagulopathy. The severe condition of the patients in this study may explain this lower success rate. Moreover, the patients described in other studies were treated during the development of the technique since these promising results, not only the indications for SAE have been extended to PPH after caesarean delivery, vaginal or cervical tears, but the severity of illness of the patients treated is more important than the patients in the preliminary studies.

### Limitations

One of the major limitation of the haemodynamic parameter is the difficulty to exactly assess the boold Loss. The use of a systematic collection bag is part of the recommendation of the french college of Obstetrics and Gynecology. However it probably underestimate the real blood loss. This study reports results of SAE performed by 7 different radiologists, compared with only 4 in our previous report [16], [18]: the reproducibility of the technique may also be responsible for the lower success rate and this matter deserve an evaluation, the number of cases in this study was not enough to reach statistical significance regarding clinical performances of the different radiologist. Most of the patients of the current study were transferred from other obstetrics units, because of the severity of their illness. This might have affected their outcome and bias the outcome of SAE in this study. However the organization of peri-natal care worldwide will not allow the setting of technical facilities for SAE in all hospitals, and patient's transfer will therefore be a necessity in most cases.

Vaginal delivery was associated with a higher success rate than was caesarean delivery (77.7% and 47.5% respectively, p<0.05). Of the causes of PPH, uterine atony was associated with the highest success rate (88.6%). Vaginal or cervical tears were associated with a trend towards SAE failure (p = 0.06). Shock was also associated with a significantly lower success rate: SAE failed in 28 of 72 (38.3%) patients with shock. Disseminated intravascular coagulopathy was not correlated with SAE outcome.

Severe PPH is a complex situation, often involving a combination of characteristics (mode of delivery, PPH causes, and haemodynamic and coagulation status). Accordingly we conducted a multivariate analysis to identify independent factors that may influence SAE outcome.

Uterine atony remains an independent predictor of SAE success in the multivariate analysis, with an odds ratio of 4.13 (95% CI, 1.35–12.6). Caesarean delivery was correlated with poor results, with an odds ratio of 0.16 (95% CI, 0.04–0.5). Surgical methods, in particular uterine artery ligation, have been shown to be successful and may thus be more appropriate for PPH after caesareans [3], [13], [19]. The causes of PPH after caesarean are different from causes after vaginal delivery: for example, only 22.7% of the PPH in the caesarean group were due to uterine atony, compared with 48.1% in the vaginal delivery group (p = 0.03). Similarly, 40.9% of the haemorrhages after caesareans were due to abnormal placentation 40.9% but only 8.6% for the vaginal delivery group (p<0.003). These differences in the causes of PPH can explain the discrepancy of the oucome of SAE.

Shock was also a negative predictor of SAE success: the OR was 0.21 (95% CI, 0.07–0.6). Although SAE can be used in haemodynamically unstable patients as surgery can be more challenging for these patients, anything that may delay treatment and foreclose options for these patients should be avoided. Emergency hysterectomy must always be considered an option in this life-threatening situation.

We reported two deaths in this study. The causes of PPH in those cases were surgical (one occurred after a caesarean delivery and the other was caused by genital tears. As the case descriptions make clear, these patients were in extremely grave condition at the time of the SAE. The first patient stopped bleeding after the SAE, but the second required a hysterectomy. The procedure itself did not change these patients' outcome. Both, however, were transferred to our institution for SAE, and we must wonder whether this medical transfer was justified, or if it delayed optimal surgical management.

Recent studies report a return of normal menses and preservation of future fertility with successful uneventful pregnancies after SAE [20]–[22]. However, we focused on outcome of SAE in severe PPH. Our study is the first large trial to assess the results of SAE in treating life-threatening PPH in a large population. It suffers from methodological and statistical limitations because it was neither controlled nor randomised, but it is extremely difficult to conduct such a trial in this rare, life-threatening emergency situation. The success rate in a large population with extremely severe PPH is not as good as the overall rates reported in the literature. PPH due to uterine atony after vaginal delivery remains an optimal indication for SAE; caesarean deliveries and hemodynamic shock are not associated with good success rates. Several reports have confirmed the efficacy of uterine artery ligature in the treatment of PPH after caesarean [3], [13], [19]. Whether surgery should be preferred to SAE is still unknown. However it seems very difficult for both ethical and technical reasons to organize a randomised controlled trial in such a life-threatening disease that need immediate management. However as SAE does not always guarantee haemostasis in cases of severe PPH; the indications of transfer if needed for this treatment should be carefully discussed in light of the patient's haemodynamic status to minimise the risks.

In conclusion, SAE appears to be an effective method for treating PPH, but the situations in which SAE or surgical treatments such as vascular ligature or emergent hysterectomy are indicated should be clarified with a larger clinical trial.

## References

[1] Bouvier-Colle MH, Pequignot F, Jougla E (2001). [Maternal mortality in France: frequency, trends and causes].. J Gynecol Obstet Biol Reprod (Paris).

[2] Bouvier-Colle MH, Varnoux N (2001). [Maternal mortality and severe morbidity in 3 French regions: results of MOMS, a European multicenter investigation].. J Gynecol Obstet Biol Reprod (Paris).

[3] Tamizian O, Arulkumaran S (2002). The surgical management of post-partum haemorrhage.. Best Pract Res Clin Obstet Gynaecol.

[4] den Hertog CE, de Groot AN, van Dongen PW (2001). History and use of oxytocics.. Eur J Obstet Gynecol Reprod Biol.

[5] Gilbert L, Porter W, Brown VA (1987). Postpartum haemorrhage–a continuing problem.. Br J Obstet Gynaecol.

[6] Prendiville WJ (1996). The prevention of post partum haemorrhage: optimising routine management of the third stage of labour.. Eur J Obstet Gynecol Reprod Biol.

[7] Prendiville WJ, Elbourne D, McDonald S (2000). Active versus expectant management in the third stage of labour.. Cochrane Database Syst Rev.

[8] Poeschmann RP, Doesburg WH, Eskes TK (1991). A randomized comparison of oxytocin, sulprostone and placebo in the management of the third stage of labour.. Br J Obstet Gynaecol.

[9] Burchell RC (1968). Physiology of internal iliac artery ligation.. J Obstet Gynaecol Br Commonw.

[10] Evans S, McShane P (1985). The efficacy of internal iliac artery ligation in obstetric hemorrhage.. Surg Gynecol Obstet.

[11] Clark SL, Phelan JP, Yeh SY, Bruce SR, Paul RH (1985). Hypogastric artery ligation for obstetric hemorrhage.. Obstet Gynecol.

[12] Chattopadhyay SK, Deb Roy B, Edrees YB (1990). Surgical control of obstetric hemorrhage: hypogastric artery ligation or hysterectomy?. Int J Gynaecol Obstet.

[13] Vedantham S, Goodwin SC, McLucas B, Mohr G (1997). Uterine artery embolization: an underused method of controlling pelvic hemorrhage.. Am J Obstet Gynecol.

[14] AbdRabbo SA (1994). Stepwise uterine devascularization: a novel technique for management of uncontrolled postpartum hemorrhage with preservation of the uterus.. Am J Obstet Gynecol.

[15] Nizard J, Barrinque L, Frydman R, Fernandez H (2003). Fertility and pregnancy outcomes following hypogastric artery ligation for severe post-partum haemorrhage.. Hum Reprod.

[16] Pelage JP, Le Dref O, Jacob D, Soyer P, Herbreteau D (1999). Selective arterial embolization of the uterine arteries in the management of intractable post-partum hemorrhage.. Acta Obstet Gynecol Scand.

[17] Corr P (2001). Arterial embolization for haemorrhage in the obstetric patient.. Best Pract Res Clin Obstet Gynaecol.

[18] Pelage JP, Le Dref O, Mateo J, Soyer P, Jacob D (1998). Life-threatening primary postpartum hemorrhage: treatment with emergency selective arterial embolization.. Radiology.

[19] O'Leary JA (1995). Uterine artery ligation in the control of postcesarean hemorrhage.. J Reprod Med.

[20] Salomon LJ, deTayrac R, Castaigne-Meary V, Audibert F, Musset D (2003). Fertility and pregnancy outcome following pelvic arterial embolization for severe post-partum haemorrhage. A cohort study.. Hum Reprod.

[21] Descargues G, Mauger Tinlot F, Douvrin F, Clavier E, Lemoine JP (2004). Menses, fertility and pregnancy after arterial embolization for the control of postpartum haemorrhage.. Hum Reprod.

[22] Ornan D, White R, Pollak J, Tal M (2003). Pelvic embolization for intractable postpartum hemorrhage: long-term follow-up and implications for fertility.. Obstet Gynecol.

